# Nitric Oxide Synthase Type 1 Methylation Is Associated With White Matter Microstructure in the Corpus Callosum and Greater Panic Disorder Severity Among Panic Disorder Patients

**DOI:** 10.3389/fneur.2021.755270

**Published:** 2021-10-18

**Authors:** Huazhen Xu, Yuan Zhong, Shiting Yuan, Yun Wu, Zijuan Ma, Ziyu Hao, Huachen Ding, Huiqing Wu, Gang Liu, Manlong Pang, Na Liu, Chun Wang, Ning Zhang

**Affiliations:** ^1^Nanjing Brain Hospital Affiliated to Nanjing Medical University, Nanjing, China; ^2^The First Affiliated Hospital With Nanjing Medical University, Nanjing, China; ^3^School of Psychology, Nanjing Normal University, Nanjing, China; ^4^Jiangsu Key Laboratory of Mental Health and Cognitive Science, Nanjing Normal University, Nanjing, China; ^5^School of Psychology, South China Normal University, Guangzhou, China; ^6^Cognitive Behavioral Therapy Institute of Nanjing Medical University, Nanjing, China; ^7^Functional Brain Imaging Institute of Nanjing Medical University, Nanjing, China

**Keywords:** panic disorder (PD), nitric oxide synthase type I, corpus callosum (CC), DNA methylation, diffusion tensor imaging

## Abstract

**Objectives:** Methylation of the neuronal nitric oxide synthase (*NOS1*/*nNOS*) gene has recently been identified as a promising biomarker of psychiatric disorders. *NOS1* plays an essential role in neurite outgrowth and may thus affect the microstructure development of white matter (WM) in the corpus callosum (CC), which is known to be altered in panic disorder (PD). We examined the relationship between *NOS1* methylation, WM tracts in the CC, and symptoms based on this finding.

**Methods:** Thirty-two patients with PD and 22 healthy controls (HCs) were recruited after age, gender, and the education level were matched. The cell type used was whole-blood DNA, and DNA methylation of *NOS1* was measured at 20 CpG sites in the promoter region. Although 25 patients with PD were assessed with the Panic Disorder Severity Scale (PDSS), diffusion tensor imaging (DTI) scans were only collected from 16 participants with PD.

**Results:** We observed that the PD group showed lower methylation than did the HCs group and positive correlations between the symptom severity of PD and methylation at CpG4 and CpG9. In addition, CpG9 methylation was significantly correlated with the fractional anisotropy (FA) and mean diffusivity (MD) values of the CC and its major components (the genu and the splenium) in the PD group. Furthermore, path analyses showed that CpG9 methylation offers a mediating effect for the association between the MD values of the genu of the CC and PD symptom severity (95% CI = −1.731 to −0.034).

**Conclusions:** The results suggest that CpG9 methylation leads to atypical development of the genu of the CC, resulting in higher PD symptom severity, adding support for the methylation of *NOS1* as a future prognostic indicator of PD.

## Introduction

Panic disorder (PD) is a frequent anxiety disorder characterized by recurrent accidental panic attacks and unexpected anxiety. PD is considered to be a multifactorial disease resulting from the interactions of multiple genetic and environmental factors. According to previous studies, the 12-month prevalence rate of PD is 0.3%, and the lifetime prevalence rate is estimated at about 0.5% in China (1, 2).

The etiology, neuropathology, and pathophysiology of PD remain elusive. Extensive research has been performed to identify specific biomarkers of PD. Nitric oxide (NO) has been identified as a neuromodulator in the central nervous system (CNS). NO, formed by the enzyme NOS1 and encoded by the *NOS1* gene, has the capacity to influence neurodevelopment and growth, the plasticity of synapse, and neurotransmitter release (3–6). NO also plays an important role in many behavioral domains, including aggression (7), anxiety (8), depression, and cognitive functioning (9). Of note is that NO can regulate both the glutamatergic and dopaminergic systems, which are strongly implicated in the biochemical pathology of anxiety (10).

*NOS1*, located on chromosome 12q24.21 (11), is the central source of NO in the CNS (12, 13). Increasing evidence suggests that *NOS1* is implicated in multiple complex neurobiological mechanisms in neuropsychiatric disorders (14–17). For example, Zhou et al. have studied *NOS1* and its signal mechanism involved in the pathophysiology of anxiety (18).

Additionally, Kurrikoff et al. demonstrated that females with a short *NOS1* ex1f-VNTR allele had higher anxiety scores than did females homozygous for the long alleles when confronted with environmental adversity (15). A recent report by Sarginson et al. with large sample sizes corroborated this finding (19). Thus, exploring DNA methylation may be able to provide a comprehensive view of both the genetic and environmental risk factors for PD. Existing evidence shows that epigenetic modification of *NOS1* caused by DNA methylation is related to gene expression and that these *NOS1* modifications can affect the generation and bioavailability of NO (20–23). These findings provide preliminary evidence that *NOS1* methylation is an indicator of abnormal NO signaling. Also, considering that NO plays an essential role in the neuron and neurite outgrowth in the brain (24–28), this suggests potential influences of *NOS1* methylation on brain development through white matter (WM) outgrowth and neurogeny.

In the “fear network model” for PD, the important fear network regions are connected by the corpus callosum (CC) in the bilateral sides (29). The CC is an area dedicated to attention, physical complaints, and related anxiety (30). Structural abnormalities in the CC may predispose patients with PD to panic attacks because of the uneven distributions of the fear network on each side. Thus, the CC likely plays a key role in the pathophysiology of PD (31). Evidence from previous diffusion tensor imaging (DTI) studies not only supports the notion that the CC may play a critical role in PD (32–34) but also revealed an association between altered WM connectivity in PD and dysfunctional clinical symptoms. In particular, anxiety symptoms appear to impact the human WM microstructure, leading us to investigate whether epigenetic modifications (i.e., DNA methylation) contribute to these WM differences (35). DNA methylation provides tools to better understand the impact of PD symptoms on the molecular pathways in PD patients. These markers may provide insights into the potential molecular mediating pathways contributing to the neurodevelopmental effects associated with PD symptoms. Consequently, *NOS1* methylation may influence the WM microstructure of CC development in the brain, but this is yet to be investigated.

Therefore, the present study aimed to identify *NOS1* methylation and the pathways involved in WM development in PD patients. We examined the levels of *NOS1* methylation between patients with PD and healthy controls (HCs) and how the levels of DNA methylation related to the WM microstructure in the CC. We hypothesized that the level of *NOS1* methylation would differ between the two groups and are related to the WM microstructure in the CC. This study was expected to contribute to the early intervention and treatment in PD.

## Materials and Methods

### Participants

A total of 54 participants were recruited from the Nanjing Brain Hospital affiliated with Nanjing Medical University between August 2014 and July 2018. Thirty-two adult patients were diagnosed with PD by an experienced psychiatrist conforming to the Diagnostic and Statistical Manual of Mental Disorders (DSM-IV) criteria. In order to reduce the probability of diagnostic errors, all patients were evaluated with the Mini-International Neuropsychiatric Interview (MINI) by two resident physicians. Patients were also assessed with the Hamilton Anxiety Rating Scale (HAMA) (36) and the Panic Disorder Severity Scale (PDSS) (37, 38) on the same day. Magnetic resonance imaging (MRI) and peripheral blood collection were performed at the Nanjing Brain Hospital within 7 days. A deputy chief physician supervised the whole procedure. Twenty-two age- and sex-matched HCs were consecutively recruited from the Nanjing Brain Hospital *via* word of mouth and public advertisements according to the inclusion and exclusion criteria. HAMA was also used to assess the anxiety levels for HCs.

#### Inclusion and Exclusion Criteria

In the PD group, the inclusion criteria were as follows: (1) a primary diagnosis of PD by an experienced psychiatrist conforming to the DSM-IV criteria; (2) confirmation of PD diagnosis using MINI; (3) age 18–55 years; and (4) right-handed and masterful at completing all examinations. The exclusion criteria were as follows: (1) any neurological disorders or other psychiatric disorders and major physical or infectious diseases; (2) any comorbid mental illness such as depression, generalized anxiety disorder, bipolar disorder, obsessive–compulsive disorder, schizophrenia, alcohol addiction, social phobia, or eating disorder; (3) history of psychotherapy within 6 months of study enrollment; (4) inability to complete MRI; and (5) pregnancy and lactation.

The inclusion criteria for the HCs were as follows: (1) age 18–55 years; (2) HAMA total score ≤7; and (3) right-handed and masterful at completing all examinations. The exclusion criteria were: (1) any neurological disorders or other psychiatric disorders and major physical or infectious diseases; (2) history of mental illness or family history; (3) pregnancy and lactation; (4) history of psychotherapy within 6 months of study enrollment, and (5) inability to complete MRI.

### Analysis of *NOS1* DNA Methylation

The cell type used was whole-blood DNA, and the DNA methylation of *NOS1* was measured at 20 CpG sites in the promoter region. Specifically, the genomic regions of interest (ROIs) were analyzed with the geneCpG software and the methylation patterns determined with bisulfite sequencing (see Supplementary Table 1). Firstly, the PCR primer pairs were designed from bisulfate-converted DNA with the Methylation Primer software (see Supplementary Table 2). Genomic DNA was then extracted from frozen samples using a Genomic Tip-500 column (QIAGEN, Valencia, CA, USA) according to the manufacturer's protocol, and sulfurous acid was obtained from sulfite using the EZ DNA Methylation™-GOLD Kit (Zymo Research, Irvine, CA, USA). After PCR amplification (HotStarTaq polymerase kit; TAKARA, Tokyo, Japan) and construction of the library, the samples were sequenced using a paired-end sequencing protocol according to the manufacturer's guidelines (Illumina MiSeq Benchtop Sequencer, San Diego, CA, USA) (39). Normality of the continuous variables was performed with Shapiro–Wilk's test. Statistics were performed using *t*-tests and ANOVA.

### MRI Acquisition and Processing

The MRI data (3.0T Siemens Medical System scanner, Nanjing Brain Hospital) were obtained using a diffusion tensor echo-planar pulse sequence [matrix = 128 × 128, field of view (FOV) = 240 mm × 240 mm, slice thickness = 3 mm with no interslice gap, repetition time (TR) = 6,600 ms, and echo time (TE) = 93 ms]. For the DTI, the diffusion sensitization gradients were applied in 30 non-collinear directions (*b* = 1,000 s/mm^2^), together with non-diffusion-weighted acquisition (*b* = 0). During the scan (240 volumes), each participant was instructed to remain motionless and to keep the eyes closed. Foam pads and earplugs were used to reduce head movement and noise.

DTI data were processed using the pipeline for analyzing brain diffusion images (PANDA; http://www.nitrc.org/projects/panda) implemented in MATLAB (40). Firstly, all files in Digital Imaging and Communications in Medicine (DICOM) format were converted into Nifti format by MRIcron. Subsequently, using the bet command of FSL (41), the non-diffusion b0 image was used to estimate the brain mask. The purpose of the brain mask was to aid in determining the boundaries of the three dimensions of the brain. Any distortions of the diffusion-weighted images (DWI) caused by eddy current or simple head movement during scanning were corrected by registering the DWI to the b0 image through affine transformation. Then, voxel-wise calculations of the tensor matrix and the calculated diffusion tensor (DT) metrics were estimated, including fractional anisotropy (FA) and mean diffusivity (MD). MD represents the intensity of the diffusion, while FA represents the direction of water diffusion in the brain, both of which indicate the organizational and microstructural integrity of the brain WM tracts. By executing the first command of FSL, non-linear registration was performed on the individual FA and MD images in local space, which were then imported into the FA and MD templates in the Montreal Neurological Institute (MNI) space. We then resampled the diffusion metric (FA and MD) images into the MNI space with a custom spatial resolution (1 × 1 × 1 mm). Finally, the FA and MD images were smoothed with a full width of 6 mm at half of the maximum Gaussian filter.

### Regions of Interest

To explore the relationship between structural changes and the clinical symptoms in PD patients, we selected the CC and its major components (genu, body, and splenium) as ROIs. The CC mask contained in MRIcron (JHU ICBM-DTI-81 WM labels atlas) (42) included the genu, body, and splenium of the CC. The regional diffusion metrics, FA and MD, were calculated by averaging the values within each region of the CC in DPABI (43). Then, the data were statistically analyzed with SPSS 22.0.

### Statistical Analysis

Demographic and clinical characteristics were evaluated using chi-square and *t*-tests. Standard statistical protocol was used for descriptive statistics, followed by the Shapiro–Wilk's test (see Supplementary Table 3) to establish differences between the two groups. To investigate region-specific correlations between the PDSS scores and CpG4,9 methylation, we performed Pearson's correlation analysis using SPSS 22.0. Then, the CpG9 site related to the clinical symptoms was further evaluated using Spearman's correlation analysis to examine its relationship with the FA and MD values of the ROIs. Simple mediation analyses were performed using PROCESS, and age, gender, and education were included as covariates in the model. PROCESS is based on a regression-based path analysis framework and incorporates mediation and reconciliation into a conditional process model (44). All analyses were based on 5,000 bootstrap samples. When the CI did not include zero, an indirect effect was considered significant.

## Results

### Sample Characteristics

From the 40 PD patients and 40 HCs who were invited, 32 PD patients and 22 HCs met the study criteria and completed the assessment. Table 1 compares the socio-demographic and clinical characteristics of the 32 patients with PD (age ± SD = 33.1 ± 7.4, 16 males) and the 22 controls (age ± SD = 33.3 ± 7.2, 12 males). Patients and controls did not differ with regard to age, sex, or education (all *p* > 0.05) (Table 1). The HAMA total scores were significantly different between patients with PD vs. HCs (Table 1). Since seven patients with PD did not fully complete the PDSS or clinical measures, the numbers varied slightly in different categories. The mean PDSS score in patients was 14.8 (*n* = 25, SD = 5.7), ranging from 8 to 22. DNA methylation data were available for all 54 participants, and neuroimaging data were available for 16 participants with PD.

**Table 1:** Comparison of the demographic and clinical variables between PD patients and HCs.

	**PD patients (*****n*** **= 32)**		**HCs (*****n*** **= 22)**		**Statistics**		
	***N***	**%**	***N***	**%**	**χ^**2**^**	***df***	***p***
Gender					0.11	1	0.74
Male	16	50.0	12	54.5			
Female	16	50.0	10	45.5			
	**Mean**	**SD**	**Mean**	**SD**	***T***	***df***	***p***
Age (years)	33.1	7.4	33.3	7.2	0.73	52	0.94
Education (years)	13.9	3.3	15.6	3.8	1.71	52	0.09
HAMA-T	20.3	7.0	2.2	1.9	−11.8	52	0.00
PDSS score^a^	14.8	3.8					

*χ^2^ and p-values were obtained with the chi-squared test. T and p-values were obtained with two-sample t-tests. PD, panic disorder; HCs, healthy controls; SD, standard deviation; PDSS, Panic Disorder Severity Scale; HAMA-T, total score of the Hamilton Anxiety Scale. ^a^Twenty-five patients with PD completed the PDSS*.

### *NOS1* Methylation Status

**Figure 1:**
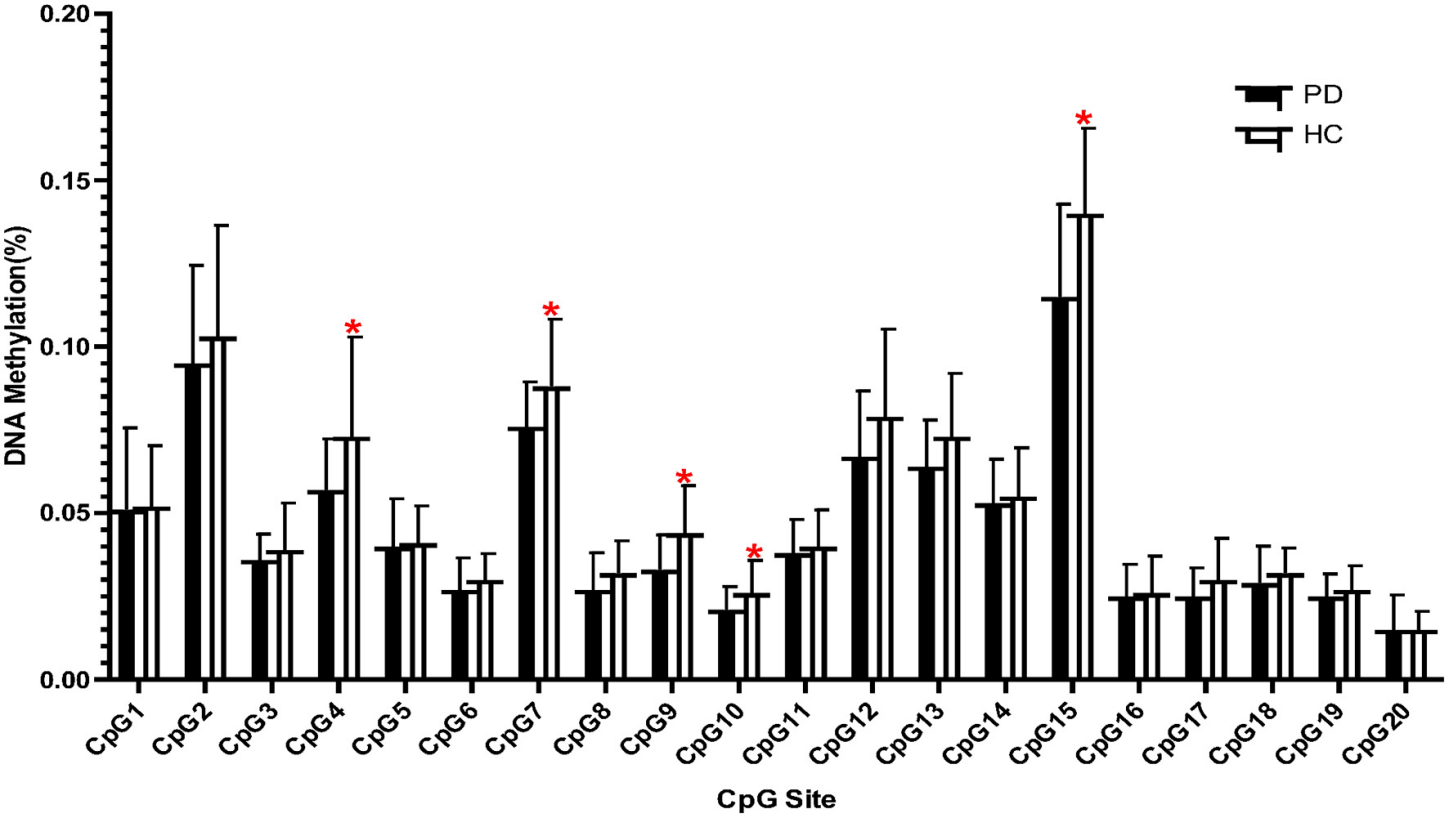
Frequency of DNA methylation at 20 CpG sites located in the promoter region of the *NOS1* gene in patients with panic disorder (PD) and in healthy controls (HCs). *p < 0.05.

We compared the methylation of each CpG unit in the PD (*n* = 32) and HC (*n* = 22) groups using *t*-tests (Figure 1). We observed that the PD group had significantly lower methylation at CpG4, CpG7, CpG9, CpG10, and CpG15 than did the HC group (*p* < 0.05). Thus, all subsequent epigenetic analyses were carried out only for these five CpG sites.

### Correlation Between Clinical Symptoms and DNA Methylation of *NOS1*

**Figure 2:**
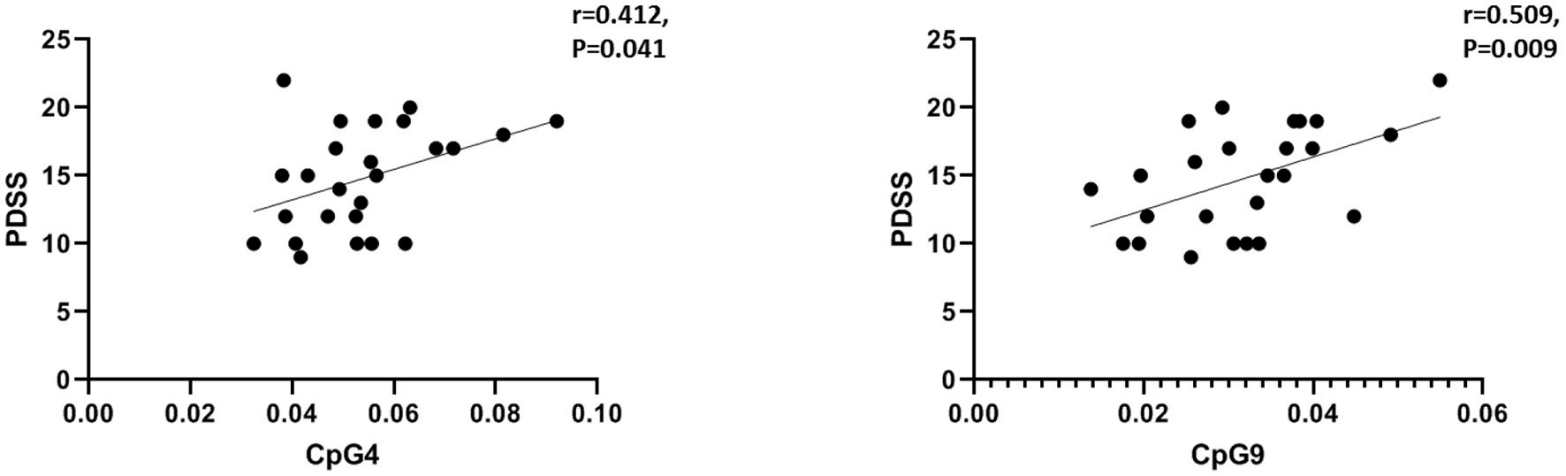
Correlations between the clinical symptoms (PDSS scores) and CpG4 and CpG9 DNA methylation in the panic disorder (PD) group. The threshold was set at a significance level of *p* < 0.05. *PDSS*, Panic Disorder Severity Scale.

We then performed Pearson's correlation analysis in the PD group to confirm the relationship between reduced methylation at five CpG sites and clinical status. A significant positive association was observed between PD symptom severity and the DNA methylation of CpG4 (*n* = 25, *r* = 0.412, *p* = 0.041) and CpG9 (*n* = 25, *r* = 0.509, *p* = 0.009) in the PD group (Figure 2), whereas no significant association was found for CpG7, CpG10, and CpG15 (*p* > 0.05).

### Correlation Between Corpus Callosum Alterations and Clinical Assessments

**Figure 3:**
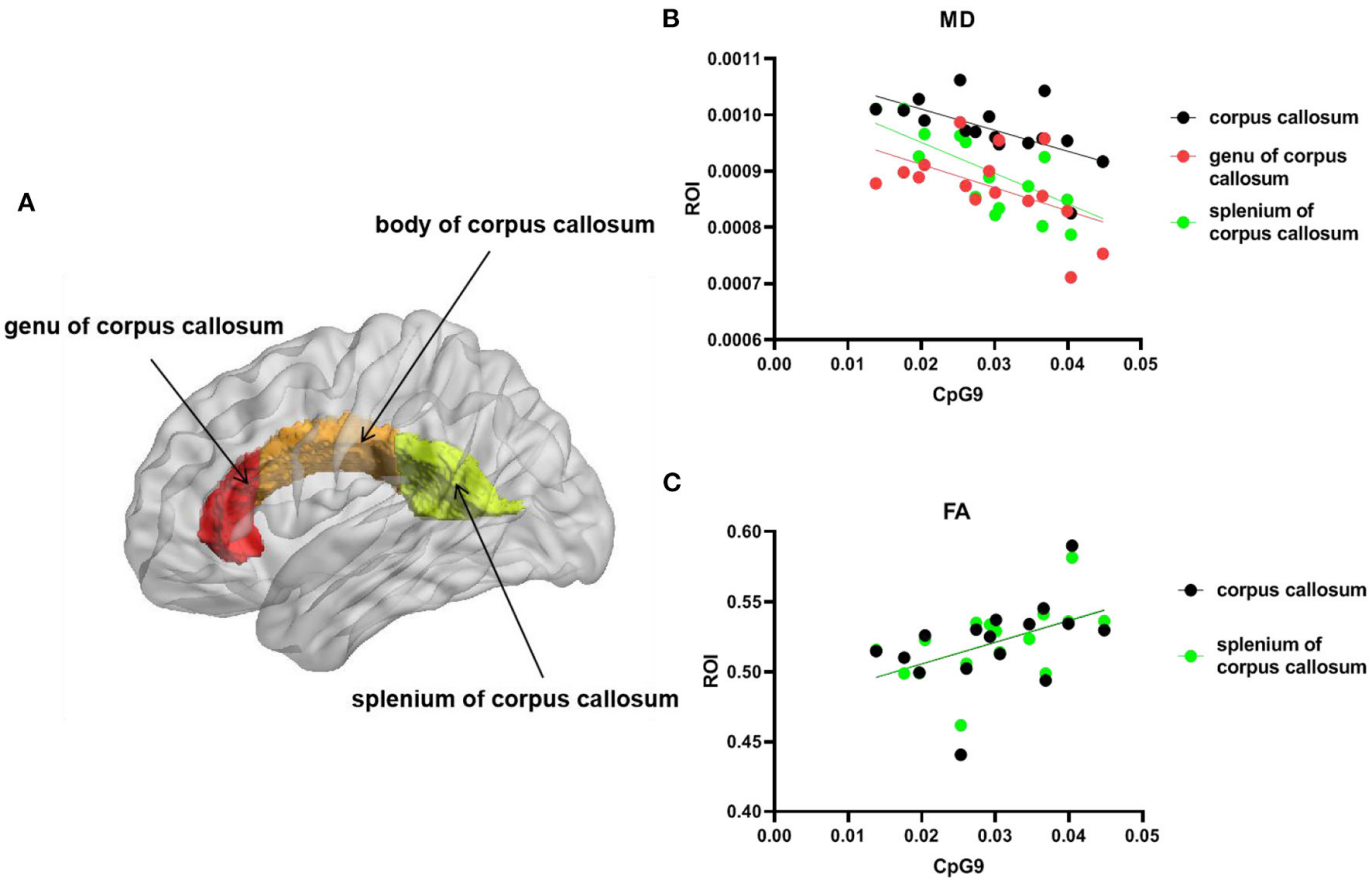
Scatter plot and *line* show the association between the degree of CpG9 methylation and the fractional anisotropy (FA) and mean diffusivity (MD) values in the region of interest (ROI) of the brain. **(A)** ROI: the total corpus callosum (CC). *Red* represents the genu, *orange* represents the body, and *green* represents the splenium of the CC. The statistical threshold for the contrasts was *p* < 0.05. **(B)** *Black circles* and *line* represent the MD values of the whole CC, the *red circles* and *line* represent the MD values of the genu of the CC, and the *green circles* and *line* represent the MD values of the splenium of the CC. **(C)** *Black circles* and *line* represent the FA values of the whole CC and the *green circles* and *line* represent the FA values of the splenium of the CC.

The FA and MD values of the bilateral CC (including three parts of it) (Figure 3A) were extracted and correlated with *NOS1* methylation. As described in the previous section and presented in Figure 2, we observed significant positive correlations between PD severity and *NOS1* methylation (at sites CpG4 and CpG9) in the PD group. We then performed Spearman's correlation analysis between CpG4 and CpG9 methylation and the FA and MD values of the CC in the PD group. As shown in Figure 3A, the results of the correlation analysis showed that the degree of CpG9 methylation was negatively correlated with the MD values in the total CC (*n* = 16, *r* = −0.700, *p* = 0.003), genu (*n* = 16, *r* = −0.532, *p* = 0.036), and the splenium (*n* = 16, *r* = −0.737, *p* = 0.002) (Figure 3B). In contrast, no significant association was found in the body of the CC (*p* > 0.05). Similarly, we found that the degree of CpG9 methylation was positively correlated with the FA values in the total CC (*n* = 16, *r* = 0.535, *p* = 0.03) and the splenium (*n* = 16, *r* = 0.647, *p* = 0.008) (Figure 3C). The effect was not statistically significant for CpG4.

### *NOS1* Epigenetic Variation as a Mediator of the Brain and Relationship With Clinical Symptoms

**Figure 4:**
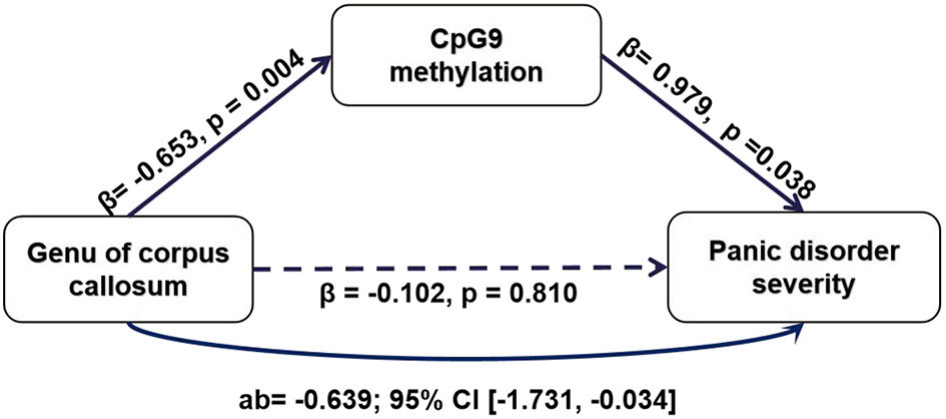
The mediating role of CpG9 methylation of *NOS1* in the relationship between the mean diffusivity (MD) values of the genu the corpus callosum (CC) and the Panic Disorder Severity Scale (PDSS) scores.

Based on the findings above, we conducted an additional path analysis to examine whether a reduced *NOS1* methylation mediated the relationship between the WM microstructure in the CC and PD symptom severity. All phenotypes were examined simultaneously in one model. As shown in Figure 4, the mediation model suggested that *NOS1* methylation at CpG9 mediated the connection between the genu of CC and PD symptom severity. The indirect effect of PD on the MD values of the genu of CC through DNA methylation was significant (β = −0.639, 95% CI = −1.731 to −0.034). The effect was not statistically significant for the other regions.

## Discussion

The current study extended the association between *NOS1* DNA methylation in PD by investigating the relationship between this novel epigenetic risk locus, clinical symptoms, and the microstructure and organization of WM tracts. We observed that the PD group showed lower *NOS1* CpG4 and CpG9 methylation than did the HC group, which led us to examine the link between CpG4 and CpG9 methylation and PD symptoms. The results showed that *NOS1* methylation at these loci had an apparent positive correlation with current PD symptoms. Finally, path analysis showed that *NOS1* methylation at the CpG9 site significantly mediated the relationship between PD and the WM microstructure in the CC. These discoveries extend previous findings by linking *NOS1* to alterations in the WM, representing a possible brain endophenotype of oxidative stress in anxiety and broadening its potential clinical service by revealing its value as a promising PD biomarker.

Although the role of *NOS1* methylation in the relationship between clinical PD symptoms and the WM microstructure has not been extensively studied, a recent study has shown that the *NOS1* ex1f-VNTR genotype is associated with clinical symptoms and a high MD value in several major WM tracts, including the genu of the CC in patients with psychotic disorders (17). This finding is in line with the notion that NO influences brain development through WM organization. Our study expanded on this by showing that WM alterations in the CC were related to *NOS1* methylation in the PD group. Our assumption that the DNA methylation of *NOS1* might add to the susceptibility of PD is verified by these results. Additionally, we found that methylation of the *NOS1* promoter was significantly reduced in the PD group and was positively associated with current PD severity. This finding is supported by a genome-wide epigenetic study that found the hypomethylation of *NOS1* in brain tissue from patients with schizophrenia (23). Additionally, previous studies have found hypomethylation of the glutamate decarboxylase 1 (*GAD1*) and monoamine oxidase A (*MAOA*) genes in PD (45, 46), which might very well describe a compensatory mechanism to counteract a genetically driven reduction of *NOS1* expression in the prefrontal cortex. Thus, our results of a positive correlation between the methylation of *NOS1* and the PDSS scores are in line with these findings because the reduction in methylation can be thought to counteract the genetically driven reduced *NOS1* expression in PD. Methylation that occurs in the CpG islands of promoter regions usually suppresses gene expression (47); therefore, a reduced methylation of *NOS1* could be associated with increased *NOS1* gene expression. NOS1, known as one of the NOS isoforms that produce the signaling molecule NO (48–50), has convincingly been linked to anxiety-like behavior (9, 51, 52) in rodents. In contrast, very few works in humans have found nitrinergic system involvement in PD. Two studies indicated that the levels of NO in the blood refer to diurnal changes in patients with PD (53, 54). Overall, NO is strongly related to the dopamine system and serotonergic neurotransmission, which offers a rationale for the participation of NO in PD.

Abnormal WM integrity in the CC in anxiety disorders has been reported in several DTI studies (29, 55, 56). The CC is a non-uniform aggregation of nerve fibers below the cortex next to the longitudinal fissure that works to integrate motor, emotional, and cognitive functions (57). The results of the structural ROI analysis revealed a correlation between a reduced *NOS1* methylation and the FA and MD values in the CC in the PD group. As is known, MD represents the average molecular motion, and both the size and integrity of cells can affect it. FA is a measure of axonal integrity and is closely associated with fiber integrity (58). Consistent with previous evidence for a strong genetic influence on the organization and microstructure of WM tracts (59), the negative association between the MD values in the CC and CpG9 methylation, as well as the positive association between the FA values in the CC and the CpG9 methylation in our study, suggested a directional influence of *NOS1* on neurite outgrowth. For example, lower FA values or higher MD values can be an indicator of axonal density reduction or axonal mutation. Based on these findings, the hypomethylation of CpG9 may have led to the modulation of NOS signaling by affecting the *NOS1* gene transcription and expression through aberrant outgrowth and organization of the WM microstructure during early brain development. There are many *NOS1* gene products that have not been widely studied, so the precise mechanisms by which *NOS1* may influence the CC are unknown. Moreover, no neuroanatomical structure generating NO in a centralized manner has been defined since every NO-producing neuron is independent and synthesizes NO in a non-synchronous way (60). Hence, NO has different effects in the different brain regions and might even be antagonistic on the behavioral level.

Given that DNA methylation may represent a key link between external environmental factors and persistent phenotypic changes, our work provides the first evidence for a mediating role of methylation in NOS signaling in brain development in patients with PD. Although we could not clarify the causality in this cross-sectional study, path analysis showed that *NOS1* methylation significantly mediated the association between the MD values of the genu of the CC and clinical symptom severity in PD. There was no similar association for the other components in the model, which suggests that the genu of the CC might be more sensitive to changes in WM microintegrity in PD patients. Aboitiz et al. found that thin fibers are most dense in the genu of the CC (61), indicating a better capacity for interhemispheric transfer. Laitinen et al. reported that psychiatric patients with symptoms of anxiety and tension could immediately benefit from high-frequency electrical stimulation in the genu of the CC (62). These findings highlight the importance of the genu of the CC and its prominent role in the severity of PD symptoms. Furthermore, we found a mediating effect of *NOS1* hypomethylation on the relationship between the genu of the CC and the severity of symptoms in PD, while no direct relationship was found between the two. One of the possible reasons for the relevance of the genu of the CC not being observed with the PDSS may be the role negative life events play in PD. Several studies found that early-life stress was associated with increased symptom ratings and meaningful alterations in the CC (63–66). Moreover, DNA methylation has also been regarded as a gene × environment interaction biomarker (46, 67–69)). Thus, the DNA methylation mechanism may explain why adversity in early life causes biological changes in psychiatric disorders. Taken together, these findings suggest that the aberrant microstructure of the WM in the genu indirectly affects the severity of PD symptoms, mediated by the lower *NOS1* methylation expressed in the CC.

Several limitations should be noted in our study. Firstly, our results are limited given the relatively small sample size in a single center and the cross-sectional nature of our data, so potential selection bias may exist. We did not have the statistical power to draw firm conclusions on the direction of the proposed effects. Secondly, we were unable to assess gene expression and could not evaluate its relationship with the methylation changes. Thirdly, peripheral blood may not necessarily reflect the levels of DNA methylation in the CNS despite previous evidence suggesting that the DNA methylation patterns in peripheral blood cells and several brain areas are highly comparable (70, 71). Finally, confirming the mechanism of epigenetic changes and neural integrity implicated in traumatic stress may need more prospective studies.

In conclusion, these findings extend our understanding of the effects of the increased underlying neuropathological basis on psychiatric disorders by identifying *NOS1* methylation as a potentially valuable blood biomarker for WM microstructure in psychiatrically relevant brain regions, which ultimately could be applied to early intervention and treatment. Furthermore, our results provide new insights into a neurobiological pathway from *NOS1* epigenetic variation, which may confer increased susceptibility to PD.

## Data Availability Statement

The datasets presented in this article are not publicly available due to privacy and ethical restrictions. Requests to access the datasets should be directed to the corresponding author Chun Wang, fm51109@163.com.

## Ethics Statement

The studies involving human participants were reviewed and approved by the Ethics Committee of the Nanjing Brain Hospital, affiliate of Nanjing Medical University. The patients/participants provided their written informed consent to participate in this study.

## Author Contributions

HX made substantial contributions to initiate, design the study, and wrote the manuscript. HX analyzed and interpreted the data by consulting YZ who was professional with a strength in fMRI, as well as senior fellow apprentice YW and ZM who ever made a lot of contribution on collection of participants. SY, ZH, MP, GL, HD, and HW made substantial contributions in collection of image data. HX revised it critically for important intellectual content with suggestions from all authors. CW led the research training, along with NL, NZ, and YZ provided editing and writing assistance. They have approved the final version to be published. HX agree to be accountable for all aspects of the work in ensuring that questions related to the accuracy or integrity of any part of the work are appropriately investigated and resolved. All authors contributed to the article and approved the submitted version.

## Funding

The study was supported by the following funding sources: National Natural Science Foundation of China (81971289, 81901390, 81871344); Natural Science Foundation of Jiangsu Province (BK20191369); the Natural Science Foundation of the Higher Education Institutions of Jiangsu Province, China (18KJB190003); Key research and development program (Social Development) project of Jiangsu province (BE2019609).

## Conflict of Interest

The authors declare that the research was conducted in the absence of any commercial or financial relationships that could be construed as a potential conflict of interest.

## Publisher's Note

All claims expressed in this article are solely those of the authors and do not necessarily represent those of their affiliated organizations, or those of the publisher, the editors and the reviewers. Any product that may be evaluated in this article, or claim that may be made by its manufacturer, is not guaranteed or endorsed by the publisher.

## References

[1] Huang Y Wang Y Wang H Liu Z Yu X Yan J. Prevalence of mental disorders in China: a cross-sectional epidemiological study. Lancet Psychiatry. (2019) 6:211–24. 10.1016/S2215-0366(18)30511-X 30792114

[2] Xiang YT Zhang Q Wang G Zeng LN Ungvari GS. Prevalence of mental disorders in China. Lancet Psychiatry. (2019) 6:467–8. 10.1016/S2215-0366(19)30128-2 31122475

[3] Arnold WP Mittal CK Katsuki S Murad F. Nitric oxide activates guanylate cyclase and increases guanosine 3':5'-cyclic monophosphate levels in various tissue preparations. Proc Natl Acad Sci U S A. (1977) 74:3203–7. 10.1073/pnas.74.8.3203 20623 PMC431498

[4] Garthwaite J. Glutamate, nitric oxide and cell-cell signalling in the nervous system. Trends Neurosci. (1991) 14:60–7. 10.1016/0166-2236(91)90022-M 1708538

[5] Vincent SR. Nitric oxide: a radical neurotransmitter in the central nervous system. Prog Neurobiol. (1994) 42:129–60. 10.1016/0301-0082(94)90023-X 7480785

[6] Yun HY Dawson VL Dawson TM. Neurobiology of nitric oxide. Crit Rev Neurobiol. (1996) 10:291–316. 10.1615/CritRevNeurobiol.v10.i3-4.20 8978984

[7] Nelson RJ Trainor BC Chiavegatto S Demas GE. Pleiotropic contributions of nitric oxide to aggressive behavior. Neurosci Biobehav Rev. (2006) 30:346–55. 10.1016/j.neubiorev.2005.02.002 16483891

[8] Gulati K Rai N Ray A. Nitric Oxide and Anxiety. Vitam Horm. (2017) 103:169–92. 10.1016/bs.vh.2016.09.001 28061970

[9] Wultsch T Chourbaji S Fritzen S Kittel S Grunblatt E Gerlach M. Behavioural and expressional phenotyping of nitric oxide synthase-I knockdown animals. In: Neuropsychiatric Disorders An Integrative Approach, Springer, Vienna, pp. 69–85. (2007). 10.1007/978-3-211-73574-9_10 17982880

[10] Freudenberg F Alttoa A Reif A. Neuronal nitric oxide synthase (NOS1) and its adaptor, NOS1AP, as a genetic risk factors for psychiatric disorders. Genes Brain Behav. (2015) 14:46–63. 10.1111/gbb.12193 25612209

[11] Kishimoto J Spurr N Liao M Lizhi L Emson P Xu W. Localization of brain nitric oxide synthase (NOS) to human chromosome 12. Genomics. (1992) 14:802–4. 10.1016/S0888-7543(05)80192-2 1385308

[12] Blum-Degen D Heinemann T Lan J Pedersen V Leblhuber F Paulus W. Characterization and regional distribution of nitric oxide synthase in the human brain during normal ageing. Brain Res. (1999) 834:128–35. 10.1016/S0006-8993(99)01444-4 10407101

[13] Yun HY Dawson VL Dawson TM. Nitric oxide in health and disease of the nervous system. Mol Psychiatry. (1997) 2:300–10. 10.1038/sj.mp.4000272 9246670

[14] Hoogman M Aarts E Zwiers M Slaats-Willemse D Naber M Onnink M. Nitric oxide synthase genotype modulation of impulsivity and ventral striatal activity in adult ADHD patients and healthy comparison subjects. Am J Psychiatry. (2011) 168:1099–106. 10.1176/appi.ajp.2011.10101446 21724667

[15] Kurrikoff T Lesch KP Kiive E Konstabel K Herterich S Veidebaum T. Association of a functional variant of the nitric oxide synthase 1 gene with personality, anxiety, and depressiveness. Dev Psychopathol. (2012) 24:1225–35. 10.1017/S0954579412000661 23062293

[16] Luciano M Houlihan LM Harris SE Gow AJ Hayward C Starr JM. Association of existing and new candidate genes for anxiety, depression and personality traits in older people. Behav Genet. (2010) 40:518–32. 10.1007/s10519-009-9326-4 20052609

[17] van Ewijk H Bralten J van Duin EDA Hakobjan M Buitelaar JK Heslenfeld DJ. Female-specific association of NOS1 genotype with white matter microstructure in ADHD patients and controls. J Child Psychol Psychiatry. (2017) 58:958–66. 10.1111/jcpp.12742 28589541 PMC5513773

[18] Zhou QG Zhu XH Nemes AD Zhu DY. Neuronal nitric oxide synthase and affective disorders. IBRO Rep. (2018) 5:116–32. 10.1016/j.ibror.2018.11.004 30591953 PMC6303682

[19] Sarginson JE Deakin JF Anderson IM Downey D Thomas E Elliott R. Neuronal nitric oxide synthase (NOS1) polymorphisms interact with financial hardship to affect depression risk. Neuropsychopharmacology. (2014) 39:2857–66. 10.1038/npp.2014.137 24917196 PMC4200496

[20] Breton CV Park C Siegmund K Gauderman WJ Whitfield-Maxwell L Hodis HN. NOS1 methylation and carotid artery intima-media thickness in children. Circ Cardiovasc Genet. (2014) 7:116–22. 10.1161/CIRCGENETICS.113.000320 24622112 PMC4008829

[21] Breton CV Salam MT Wang X Byun HM Siegmund KD Gilliland FD. Particulate matter, DNA methylation in nitric oxide synthase, and childhood respiratory disease. Environ Health Perspect. (2012) 120:1320–6. 10.1289/ehp.1104439 22591701 PMC3440108

[22] Das S Foley N Bryan K Watters KM Bray I Murphy DM. MicroRNA mediates DNA demethylation events triggered by retinoic acid during neuroblastoma cell differentiation. Cancer Res. (2010) 70:7874–81. 10.1158/0008-5472.CAN-10-1534 20841484 PMC2955783

[23] Wockner LF Noble EP Lawford BR Young RM Morris CP Whitehall VL. Genome-wide DNA methylation analysis of human brain tissue from schizophrenia patients. Transl Psychiatry. (2014) 4:e339. 10.1038/tp.2013.111 24399042 PMC3905221

[24] Candemir E Kollert L Weissflog L Geis M Muller A Post AM. Interaction of NOS1AP with the NOS-I PDZ domain: Implications for schizophrenia-related alterations in dendritic morphology. Eur Neuropsychopharmacol. (2016) 26:741–55. 10.1016/j.euroneuro.2016.01.008 26861996

[25] Chen J Zacharek A Li Y Li A Wang L Katakowski M. N-cadherin mediates nitric oxide-induced neurogenesis in young and retired breeder neurospheres. Neuroscience. (2006) 140:377–88. 10.1016/j.neuroscience.2006.02.064 16580782 PMC2791333

[26] Karson CN Griffin WS Mrak RE Husain M Dawson TM Snyder SH. Nitric oxide synthase (NOS) in schizophrenia: increases in cerebellar vermis. Mol Chem Neuropathol. (1996) 27:275–84. 10.1007/BF02815109 9147413

[27] Park SY Kang MJ Han JS. Neuronal NOS Induces Neuronal Differentiation Through a PKCalpha-Dependent GSK3beta Inactivation Pathway in Hippocampal Neural Progenitor Cells. Mol Neurobiol. (2017) 54:5646–56. 10.1007/s12035-016-0110-1 27624386

[28] Scheiblich H Bicker G. Nitric oxide regulates antagonistically phagocytic and neurite outgrowth inhibiting capacities of microglia. Dev Neurobiol. (2016) 76:566–84. 10.1002/dneu.22333 26264566

[29] Lai CH Wu YT. Fronto-occipital fasciculus, corpus callosum and superior longitudinal fasciculus tract alterations of first-episode, medication-naive and late-onset panic disorder patients. J Affect Disord. (2013) 146:378–82. 10.1016/j.jad.2012.09.022 23084185

[30] Badaruddin DH Andrews GL Bolte S Schilmoeller KJ Schilmoeller G Paul LK. Social and behavioral problems of children with agenesis of the corpus callosum. Child Psychiatry Hum Dev. (2007) 38:287–302. 10.1007/s10578-007-0065-6 17564831

[31] Gorman JM Kent JM Sullivan GM Coplan JD. Neuroanatomical hypothesis of panic disorder, revised. Am J Psychiatry. (2000) 157:493–505. 10.1176/appi.ajp.157.4.493 10739407

[32] Kim B Shin WS Kim MK Lee SH. White matter microstructural changes are associated with alcohol use in patients with panic disorder. J Affect Disord. (2016) 199:65–72. 10.1016/j.jad.2016.03.055 27085658

[33] Kim MK Kim B Kiu Choi T Lee SH. White matter correlates of anxiety sensitivity in panic disorder. J Affect Disord. (2017) 207:148–56. 10.1016/j.jad.2016.08.043 27721189

[34] Kim MS Kim YS Lee HK Lee GJ Choi CY Lee CH. Primary intracranial ectopic craniopharyngioma in a patient with probable Gardner's syndrome. J Neurosurg. (2014) 120:337–41. 10.3171/2013.10.JNS131401 24266539

[35] Dean DC Planalp EM Wooten W Kecskemeti SR Adluru N Schmidt CK. Association of prenatal maternal depression and anxiety symptoms with infant white matter microstructure. JAMA Pediatr. (2018) 172:973–81. 10.1001/jamapediatrics.2018.2132 30177999 PMC6190835

[36] Hamilton MJBJMP. The assessment of anxiety states by rating. (1959) 32:50–5. 10.1111/j.2044-8341.1959.tb00467.x 13638508

[37] Shear MK Brown TA Barlow DH Money R Sholomskas DE Woods SW. Multicenter collaborative panic disorder severity scale. Am J Psychiatry. (1997) 154:1571–5. 10.1176/ajp.154.11.1571 9356566

[38] Shear MK Rucci P Williams J Frank E Grochocinski V Vander Bilt J. Reliability and validity of the Panic Disorder Severity Scale: replication and extension. J Psychiatr Res. (2001) 35:293–6. 10.1016/S0022-3956(01)00028-0 11591432

[39] Masser DR Berg AS Freeman WM. Focused, high accuracy 5-methylcytosine quantitation with base resolution by benchtop next-generation sequencing. Epigenetics Chromatin. (2013) 6:33. 10.1186/1756-8935-6-33 24279302 PMC3907040

[40] Cui Z Zhong S Xu P He Y Gong G. PANDA: a pipeline toolbox for analyzing brain diffusion images. Front Hum Neurosci. (2013) 7:42. 10.3389/fnhum.2013.00042 23439846 PMC3578208

[41] Smith SM Jenkinson M Woolrich MW Beckmann CF Behrens TE Johansen-Berg H. Advances in functional and structural MR image analysis and implementation as FSL. Neuroimage. (2004) 23:S208–219. 10.1016/j.neuroimage.2004.07.051 15501092

[42] Desikan RS Segonne F Fischl B Quinn BT Dickerson BC Blacker D. An automated labeling system for subdividing the human cerebral cortex on MRI scans into gyral based regions of interest. Neuroimage. (2006) 31:968–80. 10.1016/j.neuroimage.2006.01.021 16530430

[43] Mori S Oishi K Jiang H Jiang L Li X Akhter K. Stereotaxic white matter atlas based on diffusion tensor imaging in an ICBM template. Neuroimage. (2008) 40:570–82. 10.1016/j.neuroimage.2007.12.035 18255316 PMC2478641

[44] Hayes A. Introduction to mediation, moderation, and conditional process analysis. J Educ Meas. (2013) 51:335–37.

[45] Domschke K Tidow N Kuithan H Schwarte K Klauke B Ambree O. Monoamine oxidase A gene DNA hypomethylation - a risk factor for panic disorder? Int J Neuropsychopharmacol. (2012) 15:1217–28. 10.1017/S146114571200020X 22436428

[46] Domschke K Tidow N Schrempf M Schwarte K Klauke B Reif A. Epigenetic signature of panic disorder: a role of glutamate decarboxylase 1 (GAD1) DNA hypomethylation? Prog Neuropsychopharmacol Biol Psychiatry. (2013) 46:189–96. 10.1016/j.pnpbp.2013.07.014 23906988

[47] Portela A Esteller M. Epigenetic modifications and human disease. Nat Biotechnol. (2010) 28:1057–68. 10.1038/nbt.1685 20944598

[48] Bredt DS Hwang PM Glatt CE Lowenstein C Reed RR Snyder SH. Cloned and expressed nitric oxide synthase structurally resembles cytochrome P-450 reductase. Nature. (1991) 351:714–8. 10.1038/351714a0 1712077

[49] Lowenstein CJ Glatt CS Bredt DS Snyder SH. Cloned and expressed macrophage nitric oxide synthase contrasts with the brain enzyme. Proc Natl Acad Sci U S A. (1992) 89:6711–5. 10.1073/pnas.89.15.6711 1379716 PMC49573

[50] Xie QW Cho HJ Calaycay J Mumford RA Swiderek KM Lee TD. Cloning and characterization of inducible nitric oxide synthase from mouse macrophages. Science. (1992) 256:225–8. 10.1126/science.1373522 1373522

[51] Schuman EM Madison DV. A requirement for the intercellular messenger nitric oxide in long-term potentiation. Science. (1991) 254: 1503–6. 10.1126/science.1720572 1720572

[52] Volke V Wegener G Bourin M Vasar E. Antidepressant- and anxiolytic-like effects of selective neuronal NOS inhibitor 1-(2-trifluoromethylphenyl)-imidazole in mice. Behav Brain Res. (2003) 140:141–7. 10.1016/S0166-4328(02)00312-1 12644287

[53] Herken H Akyol O Yilmaz HR Tutkun H Savas HA Ozen ME. Nitric oxide, adenosine deaminase, xanthine oxidase and superoxide dismutase in patients with panic disorder: alterations by antidepressant treatment. Hum Psychopharmacol. (2006) 21:53–9. 10.1002/hup.742 16329160

[54] Kaya B Unal S Karabulut AB Turkoz Y. Altered diurnal variation of nitric oxide production in patients with panic disorder. Tohoku J Exp Med. (2004) 204:147–54. 10.1620/tjem.204.147 15383695

[55] Bora E Harrison BJ Fornito A Cocchi L Pujol J Fontenelle LF. White matter microstructure in patients with obsessive-compulsive disorder. J Psychiatry Neurosci. (2011) 36:42–6. 10.1503/jpn.100082 21118658 PMC3004974

[56] Wang Y Yan FS Lian JM Dou SW. Effects of gestational magnetic resonance imaging on methylation status of leptin promoter in the placenta and cord blood. PLoS ONE. (2016) 11:e0147371. 10.1371/journal.pone.0147371 26789724 PMC4720398

[57] Aboitiz F Lopez J Montiel J. Long distance communication in the human brain: timing constraints for inter-hemispheric synchrony and the origin of brain lateralization. Biol Res. (2003) 36:89–99. 10.4067/S0716-97602003000100007 12795208

[58] Tae WS Ham BJ Pyun SB Kang SH Kim BJ. Current Clinical Applications of Diffusion-Tensor Imaging in Neurological Disorders. J Clin Neurol. (2018) 14:129–40. 10.3988/jcn.2018.14.2.129 29504292 PMC5897194

[59] Kochunov P Jahanshad N Marcus D Winkler A Sprooten E Nichols TE. Heritability of fractional anisotropy in human white matter: a comparison of Human Connectome Project and ENIGMA-DTI data. Neuroimage. (2015) 111:300–11. 10.1016/j.neuroimage.2015.02.050 25747917 PMC4387079

[60] Abkevich V Camp NJ Hensel CH Neff CD Russell DL Hughes DC. Predisposition locus for major depression at chromosome 12q22-12q23.2. Am J Hum Genet. (2003) 73:1271–81. 10.1086/379978 14606042 PMC1180393

[61] Aboitiz F Scheibel AB Fisher RS Zaidel E. Fiber composition of the human corpus callosum. Brain Res. (1992) 598:143–53. 10.1016/0006-8993(92)90178-C 1486477

[62] Laitinen LV. Stereotactic lesions in the knee of the corpus callosum in the treatment of emotional disorders. Lancet. (1972) 1:472–5. 10.1016/S0140-6736(72)90124-9 4109819

[63] De Bellis MD Keshavan MS Clark DB Casey BJ Giedd JN Boring AM. AE Bennett Research Award. Developmental traumatology. Part II: Brain development. Biol Psychiatry. (1999) 45:1271–84. 10.1016/S0006-3223(99)00045-1 10349033

[64] Jackowski AP Douglas-Palumberi H Jackowski M Win L Schultz RT Staib LW. Corpus callosum in maltreated children with posttraumatic stress disorder: a diffusion tensor imaging study. Psychiatry Res. (2008) 162:256–61. 10.1016/j.pscychresns.2007.08.006 18296031 PMC3771642

[65] Teicher MH Dumont NL Ito Y Vaituzis C Giedd JN Andersen SL. Childhood neglect is associated with reduced corpus callosum area. Biol Psychiatry. (2004) 56:80–5. 10.1016/j.biopsych.2004.03.016 15231439

[66] Teicher MH Samson JA Sheu YS Polcari A McGreenery CE. Hurtful words: association of exposure to peer verbal abuse with elevated psychiatric symptom scores and corpus callosum abnormalities. Am J Psychiatry. (2010) 167:1464–71. 10.1176/appi.ajp.2010.10010030 20634370 PMC3246683

[67] Mehta D Klengel T Conneely KN Smith AK Altmann A Pace TW. Childhood maltreatment is associated with distinct genomic and epigenetic profiles in posttraumatic stress disorder. Proc Natl Acad Sci U S A. (2013) 110:8302–7. 10.1073/pnas.1217750110 23630272 PMC3657772

[68] Smart C Strathdee G Watson S Murgatroyd C McAllister-Williams RH. Early life trauma, depression and the glucocorticoid receptor gene–an epigenetic perspective. Psychol Med. (2015) 45:3393–410. 10.1017/S0033291715001555 26387521

[69] Turecki G Meaney MJ. Effects of the Social Environment and Stress on Glucocorticoid Receptor Gene Methylation: A Systematic Review. Biol Psychiatry. (2016) 79:87–96. 10.1016/j.biopsych.2014.11.022 25687413 PMC4466091

[70] Murphy BC O'Reilly RL Singh SM. Site-specific cytosine methylation in S-COMT promoter in 31 brain regions with implications for studies involving schizophrenia. Am J Med Genet B Neuropsychiatr Genet. (2005) 133B:37–42. 10.1002/ajmg.b.30134 15635661

[71] Nohesara S Ghadirivasfi M Mostafavi S Eskandari MR Ahmadkhaniha H Thiagalingam S. DNA hypomethylation of MB-COMT promoter in the DNA derived from saliva in schizophrenia and bipolar disorder. J Psychiatr Res. (2011) 45:1432–8. 10.1016/j.jpsychires.2011.06.013 21820670

