# Supplementary material for: Nitric Oxide Synthase Type 1 Methylation Is Associated With White Matter Microstructure in the Corpus Callosum and Greater Panic Disorder Severity Among Panic Disorder Patients
Source: Front Neurol. 2021 Oct 18;12:755270. doi: 10.3389/fneur.2021.755270 (PMC8559336; doi:10.3389/fneur.2021.755270)
Supplement: Supplementary file 1 [file Data_Sheet_1.doc]

Supplementary **Table 1.**

Methylated CpG sites measured in this study.

| Gene | Position | Genomic Location* | Relative to TSS, bp |
| --- | --- | --- | --- |
| NOS1 | CpG1 | Chr12：117798172 | +1435 |
|  | CpG2 | Chr12：117798186 | +1421 |
|  | CpG3 | Chr12：117798216 | +1391 |
|  | CpG4 | Chr12：117798220 | +1387 |
|  | CpG5 | Chr12：117798242 | +1365 |
|  | CpG6 | Chr12：117798247 | +1360 |
|  | CpG7 | Chr12：117798252 | +1355 |
|  | CpG8 | Chr12：117798257 | +1350 |
|  | CpG9 | Chr12：117798259 | +1348 |
|  | CpG10 | Chr12：117798266 | +1341 |
|  | CpG11 | Chr12：117798289 | +1318 |
|  | CpG12 | Chr12：117798322 | +1285 |
|  | CpG13 | Chr12：117798325 | +1282 |
|  | CpG14 | Chr12：117798344 | +1263 |
|  | CpG15 | Chr12：117798352 | +1255 |
|  | CpG16 | Chr12：117798370 | +1237 |
|  | CpG17 | Chr12：117798374 | +1233 |
|  | CpG18 | Chr12：117798380 | +1227 |
|  | CpG19 | Chr12：117798389 | +1218 |
|  | CpG20 | Chr12：117798391 | +1216 |

*The chromosomal location of each CpG site according to assembly GRCh37/hg19

Supplementary **Table 2.**

Primer sequences for NOS1 gene

| Gene | TSS | Primer |  |
| --- | --- | --- | --- |
| NOS1 | 117799607 | forward | GAGTGGTTTTGGAAGTTGGATT |
|  |  | reverse | TTTACRAAAAACCTCCTTACRCTCTCCT |

Supplementary **Table 3.**

NOS1 methylation in patients with PD and HCs

|  | PD (n=32) | |  | HCs (n=22) | |  | Statistics | | |
| --- | --- | --- | --- | --- | --- | --- | --- | --- | --- |
|  |  | |  |  | |  |  |  | |
|  | Mean methylation | SD |  | Mean methylation | SD |  | W | | P^a^ |
| CpG1 | 0.051 | 0.0246 |  | 0.052 | 0.0183 |  | 0.873 | | 0.001 |
| CpG2 | 0.095 | 0.0295 |  | 0.103 | 0.0334 |  | 0.973 | | 0.574 |
| CpG3 | 0.036 | 0.0077 |  | 0.039 | 0.0140 |  | 0.928 | | 0.035 |
| CpG4 | 0.057 | 0.0153 |  | 0.073 | 0.0299 |  | 0.973 | | 0.587 |
| CpG5 | 0.040 | 0.0144 |  | 0.041 | 0.0112 |  | 0.960 | | 0.280 |
| CpG6 | 0.027 | 0.0095 |  | 0.030 | 0.0078 |  | 0.977 | | 0.716 |
| CpG7 | 0.076 | 0.0134 |  | 0.088 | 0.0202 |  | 0.977 | | 0.707 |
| CpG8 | 0.027 | 0.0111 |  | 0.032 | 0.0097 |  | 0.877 | | 0.002 |
| CpG9 | 0.033 | 0.0105 |  | 0.044 | 0.0143 |  | 0.982 | | 0.865 |
| CpG10 | 0.021 | 0.0069 |  | 0.026 | 0.0098 |  | 0.979 | | 0.771 |
| CpG11 | 0.038 | 0.0101 |  | 0.040 | 0.0110 |  | 0.975 | | 0.636 |
| CpG12 | 0.067 | 0.0197 |  | 0.079 | 0.0263 |  | 0.939 | | 0.071 |
| CpG13 | 0.064 | 0.0140 |  | 0.073 | 0.0190 |  | 0.931 | | 0.042 |
| CpG14 | 0.053 | 0.0132 |  | 0.055 | 0.0146 |  | 0.961 | | 0.294 |
| CpG15 | 0.115 | 0.0278 |  | 0.140 | 0.0256 |  | 0.900 | | 0.006 |
| CpG16 | 0.025 | 0.0096 |  | 0.026 | 0.0111 |  | 0.964 | | 0.354 |
| CpG17 | 0.025 | 0.0085 |  | 0.030 | 0.0124 |  | 0.963 | | 0.335 |
| CpG18 | 0.029 | 0.0111 |  | 0.032 | 0.0075 |  | 0.971 | | 0.542 |
| CpG19 | 0.025 | 0.0068 |  | 0.027 | 0.0072 |  | 0.949 | | 0.135 |
| CpG20 | 0.015 | 0.0104 |  | 0.015 | 0.0055 |  | 0.855 | | 0.001 |

^a^ W and P value were obtained by Shapiro-Wilk's test
